# NDM-1–producing Strains, Family *Enterobacteriaceae,* in Hospital, Beijing, China

**DOI:** 10.3201/eid2002.121263

**Published:** 2014-02

**Authors:** Guang Zhou, Si Guo, Yanping Luo, Liyan Ye, Yang Song, Guangwei Sun, Ling Guo, Yong Chen, Li Han, Jiyong Yang

**Affiliations:** Chinese PLA General Hospital, Beijing, China (G. Zhou, Y. Luo, L. Ye, Y. Song, G. Sun, L. Guo, J. Yang);; Henan Provincial People’s Hospital, Zhengzhou, China (S. Guo);; Chinese PLA Institute for Disease Control and Prevention, Academy of Military Medical Sciences, Beijing (Y. Chen, L. Han)

**Keywords:** NDM-1, Enterobacteriaceae, Providencia rettgeri, Raoultella ornithinolytica, bacteria, China, New Delhi metallo-β-lactamase-1–producing strains

**To the Editor:** The prevalence of New Delhi metallo-β-lactamase-1 (NDM-1)–producing strains (family *Enterobacteriaceae*) in China remains unclear. Recently, to clarify the prevalence of *bla*_NDM-1_ in *Enterobacteriaceae* strains, we carried out retrospective surveillance for *bla*_NDM-1_ among carbapenem-resistant enterobacterial strains isolated from patients at the Chinese PLA General Hospital in Beijing. This tertiary teaching hospital has 4,000 beds and 12,000 daily outpatient visits. More than 50% of patients admitted to the hospital are from areas outside Beijing. During January 2009–June 2013, a total of 8,586 enterobacterial isolates were obtained from routine clinical samples that had been passively sent to the microbiology department. Of these, 242 (2.8%) strains exhibited resistance to carbapenems. 

In this study, we used PCR amplification to screen the carbapenem-resistant strains for the *bla*_NDM-1_ gene and other common resistance determinants. The MICs of various antimicrobial drugs were measured by E-test (AB bioMérieux, Solna, Sweden). S1 nuclease pulsed-field gel electrophoresis and Southern blot analysis were used to identify the sizes of *bla*_NDM-1_-carrying plasmids. The incompatibility (Inc) groups of the plasmids were detected by several multiplex and simplex PCRs. Multilocus sequence typing (MLST) was carried out for *Klebsiella* *pneumoniae* and *Escherichia coli* isolates, according to protocols provided on MLST websites (www.pasteur.fr/recherche/genopole/PF8/mlst/Kpneumoniae.html and http://mlst.ucc.ie/mlst/dbs/Ecoli). The transferability of plasmids was identified by conjugation experiments.

Five *bla*_NDM-1_-positive enterobacterial isolates of the following species were identified: *E. coli* (1 isolate in October2010), *K. pneumoniae* (1 isolate in August 2012), *Providencia* *rettgeri* (1 isolate in October 2012), *Enterobacter cloacae* (1 isolate in November 2012), and *Raoultella* *ornithinolytica* (1 isolate in March 2013). According to the 2013 Clinical and Laboratory Standards Institute performance standard M100-S23 (www.clsi.org/), the NDM-1-producing *K. pneumoniae* (IR5047) isolate exhibited low-level resistance to imipenem and meropenem, whereas other isolates showed high-level resistance to carbapenems. Only *E. coli* and *Providencia rettgeri,* which carry 16S rRNA methylase genes, exhibited high-level resistance to amikacin (Table). S1 nuclease pulsed-field gel electrophoresis and Southern blot analysis showed that the *bla*_NDM-1_ gene was located on plasmids of various sizes belonging to different Inc groups. The *K. pneumoniae* isolate was defined as a novel ST1240 with the allelic profile 2–1-1–1-1–3-24, and the *E. coli* isolate was identified as ST167.

**Table:** Phenotype and molecular characteristics of NDM-1–producing strains isolated from Chinese PLA General Hospital, Beijing, China, 2009–2013*

Isolate no.	Organism	Source	ST†	Size, type of bla_NDM-1_ plasmid	Prevalence of RDs	MICs, mg/L							
						CTX	FEP	TZP	IPM	MEM	ETP	AK	LVX
IR5028	*Escherichia coli*	Urine	167	≈190 kb, A/C	*bla*_TEM_, *bla*_OXA-1_,‡ *bla*_CTX_-_group 1, 2, 9,_ *aac*(*6*′)-*Ib-cr*‡*armA*‡	>256	>256	>256	>32	>32	>32	>256	>32.0
IR5047	*Klebsiella pneumoniae*	Blood	1,240	≈50 kb, X3	*bla*_TEM_, *bla*_SHV_, *bla*_OXA-1_, *qnrS*,‡*oqxAB*‡	>256	64	>256	8	6	>32	2	0.38
IR5337	*Providencia rettgeri*	Urine	NA‡	≈190 kb, A/C	*bla*_PER_, *bla*_CMY_, *mtC*‡	>256	>256	>256	>32	>32	4	>256	>32.0
IR5338	*Enterobacter cloacae*	Urine	NA	≈50 kb, X3	*bla*_SHV_,‡ *bla*_OXA-1_*,bla*_CTX-group 2_	>256	>256	>256	>32	>32	>32	1.5	2.0
IR5343	*Raoultella ornithinolytica*	Abscess	NA	≈70 kb, N	*qnrS*‡	>256	32	>256	>32	>32	>32	2	0.75

*NDM-1, New Delhi metallo-β-lactamase-1–producing; ST, sequence type; RDs, resistance determinants; CTX, cefotaxime; FEP: cefepime; TZP, piperacillin-tazobactam; IMP, imipenem; MEM, meropenem; ETP, ertapenem; AK, amikacin; LVX, levofloxacin; NA, not applicable. †The alleles at each of the multi-locus ST loci for a given isolate are combined into an allelic profile and assigned an ST designation. ‡Shown to be transferred by conjugation experiments.

In China, various *bla*_NDM-1_-carrying strains of the *Enterobacteriaceae* have been sporadically identified, including *K. pneumoniae*, *K. oxytoca*, *Escherichia coli*, *Enterobacter cloacae*, *Enterobacter aerogenes*, and *Citrobacter* *freundii* (*1**–**4*). We identified a *P*. *rettgeri* isolate and an *R*. *ornithinolytica* isolate that produced NDM-1. The *bla*_NDM-1-_positive *P. rettgeri* isolates have also been identified in Pakistan, India, Canada, and Mexico, whereas the NDM-1-producing *R. ornithinolytica* strain has only been detected in India (*5**–**9*). In this study, all 5 NDM-1–producing strains were isolated only once, and no dissemination of NDM-1–producing strains of *Enterobacteriaceae* has been found. Two strains (*K. pneumoniae* and *Enterobacter cloacae*) were isolated within 48 hours of the patient’s hospital admission, indicating the infections were imported (from Shandong and Hebei Provinces, respectively). *Escherichia* *coli*, *P*. *rettgeri,* and *R*. *ornithinolytica* were isolated 48 hours after admission of patients (from Henan and Hebei Provinces). Therefore, the patients might have acquired the NDM-1–producing *Enterobacteriaceae* strains at the hospital. 

However, the source of the *bla*_NDM-1_ determinant remains unclear. The possibility that the strains were imported cannot be excluded for the several reasons. First, examination to determine the infectious agent had not been performed for a considerable number of patients within 48 hours of their admission. Second, NDM-1–producing *Enterobacteriaceae* species have not spread in this hospital. Third, the *bla*_NDM-1_-carrying plasmids in the same Inc group and of similar size exhibited substantial differences in resistance determinants (Table), which suggests a different evolutionary origin for these isolates. In an additional survey of clinical data, we found no epidemiologic relationship between the patients who were infected by NDM-1–producing pathogens. These data suggest a sporadic pattern of NDM-1–producing enterobacteria in the hospital.

Sequencing analysis (data not shown) indicated that the *bla*_NDM-1_-carrying plasmid carried by *K. pneumoniae* (≈50 kb, IncX3) was different from the plasmid found in the *Acinetobacter* *pitti* isolate that was disseminated in an intensive care unit of the Chinese PLA General Hospital in 2008 (*10*). This finding suggests that the 2 plasmids had a different evolutionary origin. The IncX3 plasmid that we found was highly homologous (>99%) to the plasmid pNDM-HN380 (GenBank accession no. JX104760), which has been identified in several *Enterobacteriaceae* strains isolated from patients in southern China (*1*). This finding showed that the IncX3 plasmid that was 50 kb in size acted as the main factor mediating the transmission of the *bla*_NDM-1_ gene across China. IncA/C plasmids are the leading group of *bla*_NDM-1_-carrying plasmids and have been detected in *E. coli* isolated from China (*1*). In this study, *E. coli* (IR5028) and *P. rettgeri* (IR5337) carried IncA/C plasmids. However, these 2 strains exhibited diverse resistant determinants on these plasmids (Table). This observation suggested that the 2 plasmids have different integrating processes. For *R. ornithinolytica*, the *bla*_NDM-1_ gene was located on an IncN plasmid of ≈70 kb, which is very different from other plasmids.

In conclusion, we identified various NDM-1–producing enterobacterial isolates at the Chinese PLA Hospital in Beijing and the emergence of novel *bla*_NDM-1_-carrying clones among common species of *Enterobacteriaceae*, such as *K. pneumoniae* ST1240 and *E. coli* ST167. There is an urgent need for monitoring and surveillance of epidemiologic and genotypic profiles of NDM-1–producing *Enterobacteriaceae* species in China.
